# Complete Genome Sequence of Anaerostipes hadrus JCM 17467^T^

**DOI:** 10.1128/mra.00324-23

**Published:** 2023-06-13

**Authors:** Kazuyoshi Koike, Dieter M. Tourlousse, Mayu Hamajima, Yuji Sekiguchi

**Affiliations:** a Biomedical Research Institute, National Institute of Advanced Industrial Science and Technology, Tsukuba, Ibaraki, Japan; University of Rochester School of Medicine and Dentistry

## Abstract

We report a complete genome sequence of Anaerostipes hadrus JCM 17467^T^. The genome consists of a circular chromosome of 2,804,089 bp, with a G+C content of 37.3%. The genome was predicted to contain 21 rRNA genes, 65 tRNA genes, and 2,675 protein-coding sequences.

## ANNOUNCEMENT

Members of the genus *Anaerostipes* are abundant in the human gastrointestinal microflora and are considered major players associated with host health (1). In particular, members of the genus are known to fermentatively produce butyrate (2), a metabolite that has been extensively documented to promote intestinal homeostasis and has been associated with a myriad of extraintestinal functions (3). In patients with inflammatory bowel disease (IBD) and irritable bowel syndrome, *Anaerostipes* was identified as the most differentially abundant taxon, compared to healthy controls (4). Within the genus, the abundance of Anaerostipes hadrus members was shown to be reduced in patients with IBD (5), and they have also been associated with reduced risk of metabolic diseases (6). In this study, we generated a complete genome sequence of the type strain of A. hadrus (JCM 17467^T^ = ATCC 29173^T^ = VPI B2-52^T^), which was originally isolated from human feces (7), by Oxford Nanopore Technologies (ONT) sequencing.

Cells were obtained from the Japan Collection of Microorganisms (JCM) as a l-dried (liquid dried) culture and cultured anaerobically in modified yeast-casitone-fatty acids (YCFA) medium at 37°C under an atmosphere of N_2_/CO_2_ (80:20 [vol/vol]). DNA extraction was performed using the MagAttract high-molecular-weight (HMW) DNA kit (Qiagen). Libraries for sequencing were prepared with the ONT native barcoding kit 24 (SQK-NBD114.24) and sequenced on a R10.4.1 flow cell using a GridION device, with default settings (DNA translocation speed, 400 bp/s; sampling rate, 4,000 samples/s [4 kHz]). Default parameters were used for all bioinformatic analyses unless indicated otherwise below. Bases were called using Dorado v0.2.1 (https://github.com/nanoporetech/dorado) with model dna_r10.4.1_e8.2_400bps_sup@v4.0.0. Chimeric and/or concatenated reads were identified and split using Duplex Tools v0.3.1 (https://github.com/nanoporetech/duplex-tools) for four iterations with the option –allow_multiple_splits. Libraries were subsequently demultiplexed using the Guppy guppy_barcoder command v6.4.6, with concurrent trimming of barcodes (options –enable_trim_barcodes –barcode_kits SQK-NBD114-24). Reads with average quality scores of <12 and lengths of <1,000 bases were discarded using NanoFilt v2.8.0 (8), followed by extraction of a set of high-quality reads using Filtlong v0.2.0 (https://github.com/rrwick/Filtlong) with the option –mean_q_weight 10. This yielded a total of 139,012 reads (420,633,251 bases [150× coverage]), with an *N*_50_ value of 3,753 bases. The genome assembly was generated using Flye v2.9.1 (9) and polished with Medaka v1.7.3 (https://github.com/nanoporetech/medaka) using model r1041_e82_400bps_sup_v4.0.0. Completeness (99.3%) and contamination (2.0%) were evaluated with CheckM v1.1.3 lineage_wf (10). DDBJ Fast Annotation and Submission Tool (DFAST) v1.2.18 (11) was used for annotation.

The genome of A. hadrus JCM 17467^T^ consists of a single circular chromosome with a length of 2,804,089 bp and a G+C content of 37.3%. The genome was annotated to contain 7 complete rRNA operons, 65 tRNA genes, and 2,675 protein-coding sequences. We expect that availability of a complete genome sequence of A. hadrus JCM 17467^T^ may enable further insights into the role of A. hadrus in the human gastrointestinal tract.

### Data availability.

This genome sequence has been deposited in DDBJ/EMBL/GenBank under the accession number AP028031. Sequencing reads are available in the DDBJ Sequence Read Archive (DRA) under accession number DRR457762.
